# Use of Whooley Questions and GAD-2 Tools in Screening for Perinatal Mental Health: Current Expert Considerations

**DOI:** 10.3390/healthcare12242549

**Published:** 2024-12-18

**Authors:** Pietro Grussu, Melania Severo, Gianfranco J. Jorizzo, Rosa Maria Quatraro

**Affiliations:** 1 Consultorio Familiare, Italian National Health Service, Azienda ULSS 6 Euganea, 35100 Padova, Italy; 2 Department of Humanistic Studies, University of Foggia, 71122 Foggia, Italy; melania.severo@unifg.it; 3 Department of Clinical and Experimental Medicine, University of Foggia, 71122 Foggia, Italy; 4 Prenatal Medicine, Italian National Health Service, Azienda ULSS 6 Euganea, 35100 Padova, Italy; gianfrancojuric.jorizzo@aulss6.veneto.it; 5 Maternità in Difficoltà^®^, 35100 Padova, Italy; rosamaria.quatraro@gmail.com

**Keywords:** perinatal screening, anxiety, depression, mental health

## Abstract

Background/Objectives: Mental health screening of perinatal women is recommended as an essential element of routine maternity health care. Knowledge of screening conditions in healthcare settings as indicated by NICE is limited. This scoping review examines current expert considerations in the scientific literature on the use of Whooley questions and GAD-2 tools in screening for women’s perinatal mental health. Methods: The search included literature in English published prior to April 2023. Searches in PsycINFO, PubMed, Scopus, Scilit, and Google Scholar used the terms “Whooley questions” and “GAD-2”. Results: A total of 9 articles on studies using both of these tools were included. During pregnancy, rapid screening instruments permit the identification of women at increased risk for postpartum depression. They also detect continuity between depressive or anxious symptoms during pregnancy and depressive symptoms after delivery. Effectiveness compares favorably to that of some lengthier screening instruments. Rapid self-report tools appear to be most suitable for initial screening. They are indicative only of perinatal mental health and have no diagnostic value. In the individual studies considered in this review, the reliability and sensitivity of the Whooley questions and GAD-2 are unclear. Because of their self-reporting nature, outcomes may be subject to recall bias. Conclusions: Future evaluation of the instruments’ performance over the perinatal period is needed.

## 1. Introduction

Emotional and psychological well-being during pregnancy, childbirth, and the first twelve months after childbirth is generally designated as perinatal mental health [1]. During that time frame, there is a greater risk for common mental health disorders, including, but not limited to, depression and anxiety. Comorbid anxiety affects many women with depression [2].

Public health officials worldwide acknowledge the significance of the effects of perinatal depression and anxiety. This recognition has led to increased attention to the development of prevention and treatment strategies [3]. Multiple agencies recommend screening for perinatal depression and anxiety during pregnancy and postpartum as a fundamental element of quality perinatal care. Although validated screening tools are readily available, and notwithstanding the recommendations of multidisciplinary experts, the extent of screening, diagnosis, and treatment remains insufficient [4].

Improved outcomes, reduced depressive symptoms, increased remission, and/or improved response to treatment have all been predicted with the early identification of perinatal mood and anxiety disorders [5,6]. Recurrent screening on a regular basis, starting with the initial prenatal visit, repeated in the third trimester, and again in the postpartum period, promotes early intervention [7,8].

For the most part, screening for perinatal depression and anxiety is done using valid, reliable psychometric instruments. The Edinburgh Postnatal Depression Scale (EPDS) [9] and the Patient Health Questionnaire-9 items (PHQ-9) [10] are the most commonly used instruments. The EPDS and the PHQ-9 have been tested and validated as screening tools for perinatal depression in a variety of clinical and community settings worldwide [11].

For a woman’s initial primary level contact for antenatal or postnatal care, the United Kingdom’s National Institute for Clinical Excellence [12] recommends the use of the Whooley questions [13], an ultra-short screening tool composed of two binary-scoring questions.

The NICE recommendations also endorse perinatal administration of the ultra-short screening tool for Generalized Anxiety Disorder (GAD-2) [14] and other anxiety disorders. The probe for depressive and/or anxious elements can then be further broadened by administering additional instruments. A confirming clinical interview would then be carried out with patients who have screened positive.

The time-saving aspect of the ultra-short screening instruments such as the Whooley questions and GAD-2 tool can be of practical benefit in hectic primary care perinatal environments where health workers deal with sizeable caseloads and demanding work assignments.

## 2. Materials and Methods

### 2.1. Methodological Rationale

The purpose of this paper is to collect the descriptions of current expert considerations reported in studies and research that have used the Whooley questions and GAD-2 tools in screening for women’s perinatal mental health.

In our study, a scoping review with a qualitative narrative synthesis of current expert considerations was deemed to be more appropriate than a systematic review.

As such, a scoping review was undertaken, and the five-stage process methodological framework outlined by Arksey and O’Malley [15,16] was followed. Additionally, the preferred reporting items for systematic reviews and the reporting guidelines for meta-analysis extension for scoping reviews (PRISMA-ScR) [17] were used to direct the protocol.

### 2.2. Identify Research Question

The research question asked is: What are the potential and critical issues of using both Whooley questions and GAD-2 tools in screening for women’s perinatal mental health?

The PIOS protocol, a modified and adapted version of PICOS, used when no comparator is present, was employed to analyze the content of the studies [18].

P (Population): perinatal women (pregnant and postpartum) screened for mental health conditions (depression and anxiety).
I (Intervention): combined use of the Whooley questions and GAD-2 screening tools.
O (Outcome): (a) potential issues: Utility and acceptability of the combined use; (b) critical issues: ethical concerns (e.g., patient burden), false positives and false negatives, and other practical issues (e.g., difficulties in interpretation or clinical implementation).
S (Study Design): observational, exploratory, experimental, or empirical studies that include the combined use of both screening tools (Whooley questions and GAD-2).

### 2.3. Search Strategy

Literature in English published until April 2023 was included. Searches were conducted in PsychInfo, PubMed, Scopus, Scilit, and Google Scholar using the terms “Whooley questions” and “GAD-2”. Following this initial detection, a total of 24 articles on perinatal studies using both of these tools emerged.

### 2.4. Inclusion Criteria and Study Selection

Articles were included if they were: (a) empirical studies in peer-reviewed, English-language scientific journals; (b) published during the time frame outlined above; and (c) research on screening for women’s perinatal mental health in community or research settings where the authors of the manuscript reported using both Whooley questions and GAD-2 tools.

**Figure 1:**
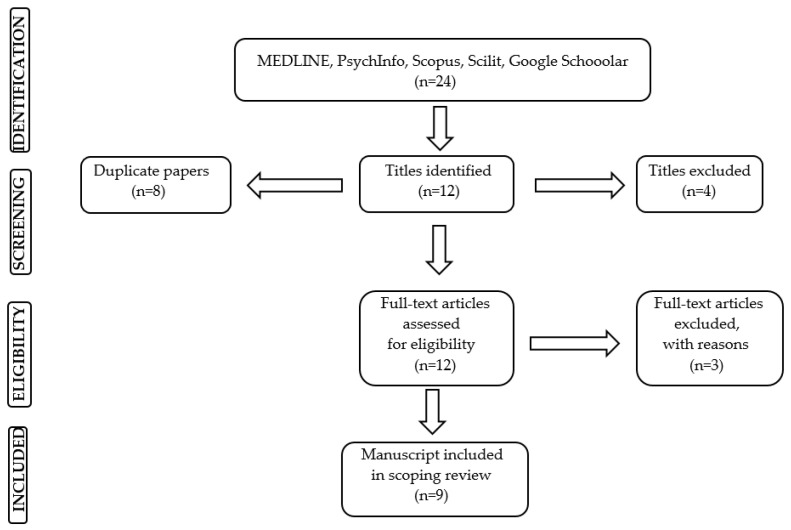
PRISMA flow chart documenting steps of the literature search.

Excluded articles were: (a) studies that were not published in the English language; (b) manuscripts present in grey literature (e.g., dissertations, abstracts from conference proceedings); and (c) studies on women’s mental health that used only the Whooley questions or only the GAD-2 tool (Figure 1).

### 2.5. Screening and Data Extraction Process

After removing duplicates, two reviewers worked separately on the process of screening article titles to minimize the risk of bias and ensure an objective analysis of the data. In the event of ambiguity or disagreements, a third reviewer was assigned to examine the discrepancies and make the final decision. This approach resulted in a high inter-rater agreement (Cohen’s κ = 0.96).

The full text of all included articles was examined by two independent reviewers who extracted the data according to a set of specific, previously agreed-upon criteria to ensure consistency and uniformity. During the extraction phase, in case of discrepancies, a third reviewer intervened to resolve the differences, consulting the original material to confirm the accuracy of the extracted data. This process ensured excellent inter-rater agreement (Cohen’s κ = 1.00).

The procedure applied during the screening and data extraction phases was also followed in the qualitative summary of the results, ensuring that any disputes were resolved impartially and minimizing the risk of errors due to divergent interpretations.

## 3. Results

The preliminary search of electronic databases yielded 24 articles. Four did not meet the inclusion criteria, eight papers were duplicates, and three articles were excluded with reasons. In all, nine articles were included in the final analysis of this scoping review. Within the References section, these nine manuscripts have been distinguished with the asterisk (*) reported at the beginning of the first author of the cited publication.

After the identification of the nine articles, two thematic sections—potential and critical issues—were expanded and are reported in full below.

### 3.1. Potential Issues

#### 3.1.1. Recommendations by Researchers in Using Both Whooley Questions and GAD-2 in the Perinatal Period

During pregnancy, the Whooley questions and GAD-2 are useful rapid screenings that allow the identification of women at increased risk for postpartum depression [19]. These same screening tools are able to detect continuity between depressive or anxious symptoms present during pregnancy and depressive symptoms present after delivery and to detect the presence of depressive and/or anxious symptoms in 95% of pregnant women who reported a prior history of mental disorders [19]. The two Whooley questions and GAD-2 item 2 turn out to be highly sensitive and specific in the antenatal period, correctly classifying anxiety and depression in 81% of cases [20]. Globally, the performance of these instruments is comparable to that of some longer screening instruments [20]. Compared with other scales, item one of the Whooley questions and item 2 of the GAD-2 proved to be the best performing ultra-short screening tools in independently predicting a major depressive episode and/or anxiety disorder diagnosis [20].

In specific contexts, the Whooley and GAD-2 questions turn out to be useful in finding that the rate of depressive and/or anxiety symptoms in women with smoking habits before pregnancy was higher than in women without smoking habits during the first trimester of pregnancy, regardless of smoking cessation [21]. Furthermore, Whooley and GAD-2 questions were able to track significantly higher anxiety-depressive symptoms in female victims of violence [22].

The Whooley and GAD-2 brevity and ease of scoring may be advantageous for use in low-resource settings with a large number of users. In addition, they could be adapted for use through online digital platforms [20].

#### 3.1.2. Recommendations by Researchers for Using Whooley Questions in the Perinatal Period

The Whooley questions may be a valid instrument when used for initial screening to detect antenatal depression. Where such an ultra-short screening instrument is used, it is advised to follow up with more in-depth screening or clinical assessment. In addition to being ultra-short, the Whooley questions, a simple binary-scoring instrument, when used by non-specialist health workers in busy, low-resource primary care settings, is possibly more feasible and acceptable than longer, Likert-type tools [20]. Whooley questions emerge as able to detect the presence of depressive symptoms in both the first trimester of pregnancy and postpartum [19]. In an antenatal setting, this instrument performs similarly to longer scales such as the EPDS [23].

Globally, the Whooley questions are the ultra-short instrument with the best results, proving to be a highly accurate instrument. This tool performs almost as well as the shortened six-item version (K6) of the Kessler Psychological Distress scale [24] and has the added benefit of having a binary scoring system, which may improve feasibility and acceptability where there are high patient volumes and low literacy levels [23]. In the detection of a Major Depressive Episode and anxiety, the Whooley questions without the third question on “need for help” turn out to have the highest specificity. The inclusion of the help question improved the performance of the Whooley and increased the sensitivity of the instrument [23]. Furthermore, clinical descriptions detected by some research protocols revealed the most serious problems and concerns in women who answered “yes” to both Whooley questions, related, in all but one case, to motherhood issues [19].

#### 3.1.3. Recommendations by Researchers for Using GAD-2 in the Perinatal Period

GAD-2 questions can detect the presence of anxiety symptoms, especially during the first trimester of pregnancy [19].

### 3.2. Critical Issues

#### 3.2.1. Limitations Reported by Researchers in Using Both the Whooley Questions and GAD-2 in the Perinatal Period

Identifying effective screening tools for use in the perinatal period for both anxiety and depression is still an unresolved challenge [23], and the reliability and accuracy of the Whooley questions and GAD-2 prove to be unclear [19].

Kujpers and colleagues [25] emphasize that the Whooley questions and the GAD-2 self-report instruments are only indicative of perinatal mental health and are without any diagnostic value. In particular, Whooley questions and GAD-2 are ineffective in identifying specific phobias in pregnancy [26]. These screening tools might be more suitable for initial screening. In fact, some researchers [23] recommended that screening with ultra-short instruments be followed by more detailed and in-depth screening to ensure more specific detection and targeted case management. Beyond follow-ups with more in-depth screening, the use of these ultra-short tools can suggest the need for an extended clinical evaluation [23]. Since the Whooley questions and GAD-2 are both self-report instruments, the results may be subject to recall bias [20]. Van Heyningen and colleagues [20] also emphasize the need for longitudinal studies that can evaluate the performance of the instruments over time or over several trimesters of pregnancy.

#### 3.2.2. Limitations Reported by Researchers in Using Whooley Questions in the Perinatal Period

Overall performance of the Whooley questions in detecting a Major Depressive Episode was marginally improved by the addition of a third item on “helping”. Sensitivity was increased by 6%, but the positive predictive value was decreased, thus increasing the number of false positives [23]. Furthermore, to avoid false positives, the Whooley questions should be pre-diagnostic and used only for screening [27].

Van Heyningen and colleagues [20] suggested including a question about suicidal thoughts to improve the identification of women at risk for suicide.

#### 3.2.3. Limitations Reported by Researchers in Using GAD-2 in the Perinatal Period

In detecting anxiety disorders, the GAD-2 had a high number of false positives [23].

In summary, the Whooley questions and GAD-2 have proven to be useful tools for the rapid screening of depression and anxiety symptoms during the perinatal period. The Whooley questions are particularly effective in identifying depression during pregnancy and the postpartum period, with good accuracy in detecting depressive and anxious symptoms in women with a history of mental health disorders. The GAD-2 has also demonstrated a good ability to detect anxiety symptoms, particularly during the first trimester of pregnancy. Both tools have shown good sensitivity and specificity, with performance comparable to longer scales but with the advantage of brevity and ease of use, making them suitable for low-resource settings with a high volume of patients. Additionally, both tools have been found useful in identifying vulnerable women, such as those with pre-pregnancy smoking habits or victims of violence.

However, some limitations related to their specificity have emerged. The Whooley questions and GAD-2 are not diagnostic tools and are not effective in identifying specific mental health disorders, such as phobias or suicidal thoughts. Furthermore, the inclusion of the third question on “need for help” in the Whooley questions improved sensitivity but also increased false positives, thereby reducing the positive predictive value. To minimize false positives, the Whooley questions should be used solely as an initial screening tool, followed by a more comprehensive assessment. Similarly, while the GAD-2 is useful for detecting anxiety, it has shown a high rate of false positives.

In general, while both tools are effective for early screening, it is important to use them in conjunction with other clinical evaluations to ensure targeted prevention and timely treatment. These tools are particularly beneficial in low-resource settings, but their lack of diagnostic specificity necessitates follow-up with more comprehensive instruments for proper case management and risk assessment.

## 4. Discussion

The contents of this scoping review were revealed by the in-depth analysis of nine peer-reviewed articles present in some of the major scientific databases. The objective was to identify the current expert considerations in the use of Whooley questions and GAD-2 tools in screening for perinatal mental health.

There were numerous considerations reported by the researchers, both in terms of the quality of the tools and their limitations and critical issues.

When used simultaneously during pregnancy and in the months following the birth of the child, the Whooley questions and GAD-2 are able to detect continuity between depressive or anxious symptoms present during pregnancy and depressive symptoms present after delivery, and to detect the presence of depressive and/or anxious symptoms in a high percentage of pregnant women who reported a prior history of mental disorders [19]. The performance of these instruments is comparable to that of some longer screening instruments, and the brevity and ease of scoring of Whooley and GAD-2 may be advantageous for use in low-resource settings with a large number of users [20]. In particular, the Whooley questions are the ultra-short instrument with the best results, which may improve feasibility and acceptability where there are high patient volumes and low literacy levels [23]. In addition to being ultra-short, the Whooley questions are a simple binary-scoring instrument that is possibly more feasible and acceptable for use by non-specialist health workers in busy, low-resource primary care settings when compared with longer, Likert-type tools [20]. Furthermore, the inclusion of the help question improved the performance of this tool and increased the sensitivity of the instrument [23]. In the antenatal period, GAD-2 question results can detect the presence of anxiety symptoms, especially during the first trimester of pregnancy [19].

Despite the presence of these positive considerations by current experts, some important limitations emerge in the use of the Whooley questions and GAD-2 in the perinatal period.

The reliability and accuracy of the Whooley questions and GAD-2 are unclear [19]. It should also be repeated that the Whooley questions and the GAD-2 are self-report instruments without any diagnostic value that are only indicative of perinatal mental health [26].

Whooley questions and GAD-2 might perhaps be more suitable for initial screening. For this reason, screening with these instruments is best followed by more detailed and in-depth screening to ensure more specific detection and targeted case management. Beyond follow-ups with more in-depth screening, positive results from the Whooley questions and GAD-2 warrant an extended clinical evaluation [23]. In terms of the individual tools taken into consideration in this scoping review, it emerges that the Whooley questions should only be pre-diagnostic and used only for screening in order to avoid false positives [27]. In detecting a Major Depressive Episode, adding the third item on helping to the two Whooley questions marginally improved overall performance but decreased the positive predictive value, thus increasing the number of false positives [23].

In detecting perinatal anxiety disorders, it should also not be overlooked that GAD-2 had a high number of false positives [23]. Furthermore, there is considerable heterogeneity in the measurements made. The studies that were analyzed differed greatly in terms of the period of assessment in pregnancy or the postpartum period, so it is difficult to compare the results that emerged. Similarly, for the most part, these are studies with single measurements over a period of time, which are probably insufficient to represent the extent of the phenomenon in the population under investigation. In consideration of the important properties of the Whooley questions and GAD-2, but also in light of their limitations, there is an emerging need for longitudinal studies that can evaluate the performance of the instruments over time or over several trimesters of pregnancy [20]. In addition, these instruments could be adapted for use through online digital platforms [20]. It should also be emphasized that when the Whooley questions and GAD-2 ultra-short screening instruments are used, it is advised to follow up with more in-depth screening or clinical assessment [20]. Furthermore, since the Whooley questions and GAD-2 are self-report instruments, the results may be subject to recall bias [20].

Using Whooley questions and GAD-2 in the perinatal period, Van Heyningen and colleagues [20] suggested including a question about suicidal thoughts to improve the identification of women at risk for suicide.

It might be interesting to further investigate the perception and acceptability of these ultra-rapid screenings among women and healthcare providers. For example, a recent study by Littlewood et al. [28] examined the acceptability of Whooley questions among both women and health workers, finding that women have difficulty responding to this ultra-rapid screening for fear of consequences and stigma. In the same study, health workers were comfortable asking Whooley questions, but only with women who had not experienced trauma or had no history of psychiatric disorders. On the other hand, in a study by Yapp and colleagues [29], most women found talking about their mental health to be acceptable. However, women with past abuse or past mental disorders showed difficulty in responding and highlighted the need for more time to talk and to receive clear, reassuring, and accurate information about mental health from health professionals. This finding raises the issue of specific training aimed at healthcare workers who conduct screenings—when they are not mental health professionals—in order to provide the right skills to give comprehensive and appropriate responses to the queries of women screened.

### 4.1. Whooley Questions and GAD-2 Key Findings

#### 4.1.1. Effectiveness for Screening

(a) Both the Whooley questions and GAD-2 have proven effective in identifying depression and anxiety during the perinatal period, particularly in women with a history of mental health disorders.
(b) The Whooley questions are especially reliable in detecting depressive symptoms both during pregnancy and postpartum.
(c) The GAD-2 tool is particularly effective in identifying anxiety, especially in the first trimester of pregnancy.
(d) These tools demonstrate high sensitivity and specificity, with performance comparable to longer screening instruments.
(e) The brevity and ease of scoring of both tools make them highly suitable for use in settings with limited resources and high patient volumes.
(f) Both tools are particularly effective in identifying vulnerable groups, such as women with pre-pregnancy smoking habits or those with a history of violence.

#### 4.1.2. Limitations

(a) The Whooley questions and GAD-2 are self-report tools that lack diagnostic value and cannot identify specific mental health disorders such as phobias or suicidal ideation.
(b) Positive results from both tools should be followed by in-depth clinical evaluation to ensure accurate diagnosis.
(c) The addition of the third item on “need for help” in the Whooley questions improves sensitivity but also increases the false positive rate.
(d) High Rate of False Positives: Both the Whooley questions and GAD-2 exhibit a high number of false positives, especially in the detection of anxiety disorders.

### 4.2. Limitations of This Manuscript

This scoping review had several limitations.

(1) The manuscripts taken into consideration in our work were limited in number. This in part confirms that, probably, many professionals and researchers involved in community-based programs for screening perinatal depression and anxiety using the Whooley questions and GAD-2 tools have not published the results in a peer-reviewed journal, thus limiting the possibility of our knowing the outcomes (see Bhat et al., [30]).
(2) The studies taken into consideration in this manuscript were not intended to verify the appropriateness and sensitivity of the double administration of Whooley questions and GAD-2. For this reason, there is limited reporting on the potential and/or critical issues of the aforementioned tools when offered simultaneously to perinatal women.
(3) The objectives of the nine peer-reviewed articles identified for this review were very different from each other. Furthermore, there was insufficient homogeneity in the times of administration of the Whooley questions and GAD-2, both during pregnancy and in the postnatal period. This discrepancy may have influenced the considerations we noted when analyzing the individual manuscripts.

## 5. Conclusions

Perinatal mood and anxiety disorders affect many women during pregnancy and postpartum, with lasting impacts on both mother and child. Systematic screening is essential for identifying women who could benefit from early mental health interventions, but it must be part of a comprehensive care plan that includes diagnosis, treatment, and follow-up. Effective management depends on integrated health information systems.

Routine screening for depression and anxiety should occur at least once during pregnancy and once postpartum in community-based programs. These settings offer frequent contact with healthcare staff, creating opportunities for early detection. Screening not only helps identify at-risk women but also serves to raise awareness, reduce stigma, and improve treatment uptake. Standardized approaches to screening in community settings are urgently needed.

The NICE guidelines recommend the Whooley questions and GAD-2 tool for screening perinatal depression and anxiety, as they can significantly reduce the need for further screening. Future research should explore the reliability of these tools and the impact of adding the “help” question. Shorter tools are particularly useful in busy medical environments, especially when non-specialist health workers are involved, as evidence shows that maternal mental health interventions can be effectively delivered by them.

However, referral pathways must be in place for women with severe conditions. For screening to be effective, it must be part of a coordinated strategy that includes training, supervision, and clear referral systems.

In practical terms, these findings emphasize that early, systematic screening combined with appropriate resources for diagnosis and treatment is crucial for improving maternal mental health and mitigating long-term risks for both mother and child.

## Data Availability

Not applicable.
